# SPARC Metrics Provide Mobility Smoothness Assessment in Oldest-Old With and Without a History of Falls: A Case Control Study

**DOI:** 10.3389/fphys.2020.00540

**Published:** 2020-06-10

**Authors:** Anelise Ineu Figueiredo, Gustavo Balbinot, Fabiane Oliveira Brauner, Aniuska Schiavo, Rafael Reimann Baptista, Aline Souza Pagnussat, Kristen Hollands, Régis Gemerasca Mestriner

**Affiliations:** ^1^Biomedical Gerontology Program, School of Medicine, Pontifical Catholic University of Rio Grande do Sul, Porto Alegre, Brazil; ^2^Neuroplasticity and Rehabilitation Research Group (NEUROPLAR), Pontifical Catholic University of Rio Grande do Sul, Porto Alegre, Brazil; ^3^KITE – Toronto Rehabilitation Institute, University Health Network, Toronto, ON, Canada; ^4^School of Health and Life Sciences, Pontifical Catholic University of Rio Grande do Sul, Porto Alegre, Brazil; ^5^Department of Physical Therapy, Federal University of Health Sciences of Porto Alegre, Porto Alegre, Brazil; ^6^School of Health Sciences, University of Salford Manchester, Salford, United Kingdom

**Keywords:** movement smoothness, functional mobility, falls, aging, oldest-old

## Abstract

Aging-related neuromuscular and neurocognitive decline induces unsmooth movements in daily functional mobility. Here, we used a robust analysis of linear and angular spectral arc length (SPARC) in the single and dual task instrumented timed up-and-go (iTUG) test to compare functional mobility smoothness in fallers and non-fallers aged 85 and older. 64 participants aged 85 and older took part in this case control study. The case group (fallers, *n* = 32) had experienced falls to the ground in the 6 months prior to the assessment. SPARC analyses were conducted in all phases of the single and dual task iTUGs. We also performed correlation mapping to test the relation of socio-demographic and clinical features on SPARC metrics. The magnitude of between-group differences was calculated using D-Cohen effect size (ES). SPARC was able to distinguish fallers during the single iTUG (ES ≈ 4.18). Turning while walking in the iTUG induced pronounced unsmooth movements in the fallers (SPARC ≈ −13; ES = 3.52) and was associated with the ability to maintain balance in the functional reach task. This information is of importance in the study of functional mobility in the oldest-old and to assess the efficacy of fall-prevention programs.

## Introduction

Aging is a natural process associated with musculoskeletal and cognitive decline (Harada et al., 2013). This process ultimately leads to reduced movement smoothness and cognitive reserve, impairing the mobility in daily life, such as walking, turning, and sitting (Sunderaraman et al., 2019). The fact that daily living activities (ADLs) require multi-tasking increases the cognitive demand and may lead to a higher risk of falls in the oldest-old (Bock, 2008; Montero-Odasso et al., 2012; Fernandez et al., 2019). Complex tasks demand higher processing speeds and greater attention, memory, and executive function, which are all affected by the natural aging process (Harada et al., 2013). The occurrence of falls may reduce life expectancy due to several secondary conditions of particular importance to the fragile elderly (Deandrea et al., 2010). Therefore, fall prevention programs are of utmost importance to the elderly, especially the fragile and the oldest-old.

To identify the above-mentioned neuromuscular and neurocognitive deficits and subsequent risk of falls, more sensitive and robust measurements of mobility smoothness during such complex tasks are warranted. Regarding complexity, the timed up-and-go (TUG) task is considered a reliable and valid test for quantifying functional mobility in the elderly—the test involves complex mobility tasks such as turning while walking or transitions from walking to sitting on a chair, which are of importance to daily living functional mobility (Podsiadlo and Richardson, 1991). While time taken in the test is the most commonly used in the test, the typical TUG lacks more specific information on movement quality, such as movement smoothness. Moreover, measures of time and transitioning angles obtained by instrumented TUG (iTUG) do not provide better fall risk classification when compared with the measures provided by typical TUG test (Jackson et al., 2018). On the other hand, unsmooth movements have been associated with poor balance and risk of falls in many populations, such as stroke survivors (Isho and Usuda, 2016; Kerr et al., 2017; Pickford et al., 2019) and people living with Parkinson’s disease (Buckley et al., 2019). Thus, movement smoothness might be of importance when performing iTUG-based mobility assessment (Weiss et al., 2011). Several approaches are used to quantify movement smoothness during walking, such as the autocorrelation coefficient (Moe-Nilssen and Helbostad, 2004) or harmonic ratios (Menz et al., 2003). Recently, the spectral arc length (SPARC), a new approach to quantifying movement smoothness, has shown great sensitivity, robustness, and reduced dependence on speed or task duration (Balasubramanian et al., 2012; Balasubramanian, 2015; Beck et al., 2018). Traditional smoothness metrics, such as the number of peaks, dimensionless, and log dimensionless jerk, are more susceptible to movement amplitude and duration (Balasubramanian, 2015). Although some of these metrics also show reduced effects of movement amplitude and duration, SPARC is less susceptible to signal noise artifacts—common in accelerometry-based measurements.

Assessing mobility smoothness using robust smoothness measures, such as SPARC, may reveal unique characteristics among populations prone to falling. In this study, we aimed to explore whether the mobility smoothness assessed by the SPARC metrics in the iTUG is different in non-institutionalized elderly aged 85 and older, with and without a history of falls in the last 6 months. In addition, we used the dual-task iTUG (a cognitive-motor task) to better understand how increasing the cognitive load might affect mobility smoothness and its relationship with the history of falls. We hypothesized that mobility smoothness, as measured by SPARC metrics, would clearly show a difference between oldest-old with and without history of falls.

## Materials and Methods

### Participants and Procedures

Participants with (case, *n* = 32) and without (control, *n* = 32) self-reported history of falls were recruited by convenience. A fall was defined as an unexpected and unexplained event in which the participant comes to rest on the ground/floor (Deandrea et al., 2010). In this study, to minimize bias in the self-reported assessment, we did not consider other types of falls. The participant who has fallen at least once in the 6 months prior to the study was considered a faller. Based on the inclusion criteria, we selected participants of any gender, aged ≥85 years, who walked independently (walking-assistant devices allowed) and understood the verbal commands necessary to adequately perform the proposed evaluation. The exclusion criteria were: uncertainty regarding the history of falls; hospitalization for more than 7 days in the previous 3 months; major unresolved orthopedic injuries; and diagnosis of neurological and/or severe respiratory, cardiovascular, visual, or auditory diseases. Data collection was as follows: (1) we explained the study aims to the participants; (2) informed consent was signed; (3) we applied the General Screening Questionnaire, Mini Mental State Examination (MMSE) (Folstein et al., 1975), Geriatric Depression Scale (GDS) (Yesavage et al., 1982)—short version, Activities Specific Balance Confidence (ABC) Scale (Powell and Myers, 1995), Falls Efficacy Scale—International (FES-I) (Yardley et al., 2005), and the International Physical Activity Questionnaire (IPAQ) adapted for the elderly (Rubio Castañeda et al., 2017). Functional reach, blood pressure, body mass, and height were also assessed. Finally, two types of TUG tests (three single task TUG and three dual task TUG trials) were performed in the following order: single task TUG—dual task TUG—dual task TUG—single task TUG—single task TUG—dual task TUG. We used three trials of each type of the TUG test to assess both possible learning and fatigue effects in the oldest-old. A similar approach was used in a previous study in multiple sclerosis (Witchel et al., 2018). The participants were seated on a standard chair (43 cm in height, without armrest) and asked to get up and walk as fast as possible. The verbal command “get up and go” was given and the participants moved from sit to stand, walked 3 m (walk 1), performed a 180° turn (turn), walked 3 m (walk 2) turned, and sat on the same chair from which they had started (turn-to-sit on the chair). The spot at which the subjects were expected to perform the 180° turn was marked by a cross on the ground using adhesive tape (30 × 30 cm). During the dual task TUG, participants followed the same above-mentioned protocol while speaking the days of the week in reverse order (e.g., Wednesday, Tuesday, Monday, Sunday, Saturday, Friday, Thursday, and so on until the test finished). The rationale for choosing this dual task was based on previous research (Barbosa et al., 2008; Fatori et al., 2015; Silva et al., 2017). They were instructed to start speaking, as quickly as-possible, while still sitting. While no penalty was applied when they had a mistake in speaking, each participant was advised the right/wrong replies would be recorded and scored. At each dual task TUG trial, the initial day of the week was changed (e.g., Wednesday, then Tuesday, and finally Monday).

### Inertial Measurement Unit (IMU)

Linear acceleration and angular velocity were measured during the TUG test using a Bluetooth-compatible inertial measurement unit (IMU; G-Walk^®^, BTS Bioengineering, MA, United States), with a sampling rate of 100 Hz. The IMU was positioned between the L5 and S1 vertebrae using an elastic belt provided by the manufacturer (Kleiner et al., 2018; Pau et al., 2018). The device has a built-in triaxial accelerometer and gyroscope. Linear acceleration (Acc L) and angular velocity (Vel A) were acquired in the vertical (V), mediolateral (ML), and anteroposterior (AP) axes. Raw acceleration and angular velocity data were extracted using the G-sensor^®^ software and exported in ASCII format.

### Mobility Smoothness Measurement and Data Analyses

Offline signal processing and analyses were performed using LabVIEW^®^ (version 8.5; National Instruments, Austin, TX, United States) custom software routines. Acc L and Vel A data were considered when the mean of a 10-frame moving window was greater than three times the SD of the initial noise (100 frames window). We developed a mathematical routine to segment the TUG test into phases. A visual inspection of the yaw and pitch angles was implemented, followed by manual cropping of each curve. The pitch angle was used to crop “sit to stand.” A combination of the yaw and pitch angles was used to detect the turn-to-sit phase, i.e., the yaw angle was used to detect the turn before sitting (≈180°), and the pitch angle to detect the trunk flexion when starting the sitting movement. The midway point of the 180° turn was detected using the yaw angle (turn phase). During the pilot data analysis, we suspected the automatic algorithm provided by BTS failed to correctly determine the duration of the TUG phases. For example, we identified the turn and turn-to-sit on the chair phases took much longer than expected considering previous trials. We understand there are two separate or combined explanations for this situation. While important papers have proposed the use of automatic algorithms in lumbar-mounted IMU (also used by BTS), they have not been specifically validated for use in people aged between 85 and 101 years old (Weiss et al., 2013; Vervoort et al., 2016; Kleiner et al., 2018; O’Brien et al., 2019). Moreover, the automatic algorithms usually consider a TUG test in which a cone is used to mark the turning point (180°). In the present study, the cone was replaced by a cross on the ground made using adhesive tape (30 × 30 cm) to minimize the influence of the visual cue in the turning performance (provided by the shape of the cone). As expected, this change increased the variability of the movement strategies adopted by the oldest-old during the turning phase, which sometimes exceeded the ability of the automatic algorithm to precisely determine the duration of the TUG phases. Thus, we developed a manual signal analysis routine in order to minimize measurement errors in the detection of TUG phases (previously published by Pinto et al., 2019). Two trained, independent assessors tested the reliability of this routine and found reliable results (Supplementary Figure S1 and Supplementary Table S1). Mean subtractions were used to remove the direct current (DC) components from raw acceleration data and whenever signal manipulations caused the drifting of the signal (Beck et al., 2018). Removing DC and drifting is important when processing acceleration measured by accelerometers, especially to remove the large DC component in the spectrum (accelerometers also pick up gravity). Subsequently, high frequencies not involved in the TUG test were removed when applying the limits of integration (0–10 Hz bandwidth). The upper and lower limits of integration were set at 0 and 10 Hz to encompass higher frequencies present during the TUG test (Pinto et al., 2019), when compared to steady-state walking (which assumed 0–5 Hz in previous studies) (Beck et al., 2018). We divided a distance of 3 m by the duration of the walk to obtain the average speed during the walking bouts (in m.s^–1^). The TUG test involves sharp turns, sit-to-stand, and turn-to-sit movements, which may induce more abrupt acceleration. We also hypothesized the oldest-old may have gait impairments, such as intermittency, i.e., as occurs in Parkinson’s disease (8 Hz freezing band). The SPARC calculation was adapted for the iTUG test, as previously described (Balasubramanian, 2015). We calculated the SPARC metrics from each trial and the average SPARC from three TUG trials using the following formula:

(1) $$S\,P\,A\,R\,C=-\int_{0}^{10}\sqrt{{\left(\frac{1}{10}\right)}^{2}+{\left(\frac{n\,o\,r\,m\,P\,S\,D\,\left(w\right)}{d\,w}\right)}^{2}}\,dw$$

where 0 and 10 Hz are the limits of integration, normPSD is the normalized power spectrum density (PSD), and dw is an infinitesimal amount of PSD frequency.

For total Acc L (Acc L total) and total Vel A (Vel A total), we used previously proposed signal processing and equations (Beck et al., 2018). Importantly, SPARC metrics assume less smooth movements are more complex in terms of their frequency composition; hence, lower SPARC values indicate less movement smoothness. The SPARC was calculated for the full iTUG as well as for the distinct phases of the test such as (i) sit-to-stand, (ii) walk 1, (iii) turn, (iv) walk 2, and (v) turn and turn-to-sit.

### Measurement of Sample Characteristics

To estimate the sample size, we used a previous study (Weiss et al., 2016). Sample size was set as 64 individuals when adopting a power of 80% and an alpha of 0.05. Thus, we decided to enroll 32 fallers and 32 non-fallers. To calculate sample size, we used an online resource from the University of British Columbia (Brant, 2017). Table 1 shows the sample characteristics.

**TABLE 1:** Sample characteristics.

	Fallers (*n* = 32)		Non-fallers (*n* = 32)	
	Mean/*n* (category)	SD	Mean/*n* (category)	SD
Age	89.9	4.4	88.6	4.1
Gender (male = 0/female = 1)	7 (0)/25 (1)	n.a	5 (0)/27 (1)	n.a
Blood pressure/systolic	127.3	11.1	127.5	11.6
Blood pressure/diastolic	77.7	12.5	74.7	8.0
Mean arterial pressure	94.2	10.4	92.3	8.1
Level of schooling/years	7.9	5.7	7.9	4.2
Marital Status (widow or not = 0/married = 1)	22 (0)/10 (1)	n.a.	24 (0)/8 (1)	n.a.
Number of medications in use	5.3	2.8	4.4	2.3
MMSE	25.8	3.6	26.8	2.5
FES-I	24.3	7.6	22.0	2.7
ABC	**73.4**	**21.1**	**82.0**	**11.6**
Ethnicity (0 = white; 1 = brown or black)	26 (0)/6 (1)	n.a.	31 (0)/1 (1)	n.a.
IPAQ (0 = sedentary; 1 = active)	**12 (0)/20 (1)**	**n.a.**	**2 (0)/30 (1)**	**n.a.**
Functional reach test (cm)	26.3	10.2	30.1	7.8
GDS depression symptoms (0 = no; 1 = yes)	20 (0)/12 (1)	n.a.	27 (0)/5 (1)	n.a.
Smoker (0 = no; 1 = yes)	31 (0)/1 (1)	n.a.	31 (0)/1 (1)	n.a.
Alcoholic drink (0 = no; 1 = yes)	26 (0)/6 (1)	n.a.	27 (0)/5 (1)	n.a.

*Independent *t*-test for quantitative variables and Chi-square with Yates’ correction for qualitative variables. Bold values denote statistical significance (*p* < 0.05).*

### Statistical Analyses

Statistical analyses were performed in GraphPad Prism^®^ version 6.01 (GraphPad Software, San Diego, CA, United States), LabVIEW^®^ and the Statistical Package for the Social Sciences (SPSS) version 17.0. We used the Shapiro–Wilk test to assess normality and applied square root and logarithmic transformations for asymmetrical variables. Nevertheless, these procedures did not work properly for many variables in this study. Thus, we performed both parametric and non-parametric analyses. Given the main findings were the same in both analyses, we decided to show them using parametric tests (the statistical plan is summarized in Supplementary Figure S2). Ordinary ANOVA using the factors falls (yes/no), trial (1, 2, 3), and task (single/dual-task TUG) was used to explore the potential between-factor interactions. We collapsed non-significant factors (i.e., trial for SPARC outcomes) and performed two-way repeated-measures ANOVA to test the factors falls (yes/no) and task (simple or dual-task TUG). Spearman’s correlation was used to test the relationship between clinical scores, demographics, and SPARC outcomes (the averages of the single and dual task TUG trials were used in the correlations). D-Cohen effect size (ES) was calculated to compare fallers and non-fallers. Qualitative data were compared using the Chi-square test with Yates’ correction and proportions. The intraclass correlation coefficient (ICC) was used to test the reliability of manual detection of the TUG phases. Data were expressed as mean and SEM and significance was set at α < 0.05.

### Ethics

This cross-sectional study was approved by the Research Ethics Committee of the Pontifical Catholic University of Rio Grande do Sul (number 099196/2017). All the participants agreed to take part and signed informed consent. We followed the Strengthening the Reporting of Observational Studies in Epidemiology (STROBE) checklist.

## Results

Table 1 describes the sample characteristics. Fallers displayed a reduced level of confidence in performing activities without losing balance (ABC score; *t* = 2.00, *p* = 0.049) and reduced level of physical activity (IPAQ; χ*^2^* = 7.40, *p* = 0.006). Supplementary Table S2 summarizes the main effects of the exploratory ANOVA.

### Greater TUG Duration for Fallers, but the Dual Task Affected Walking and Turning in Both Groups

**FIGURE 1:**
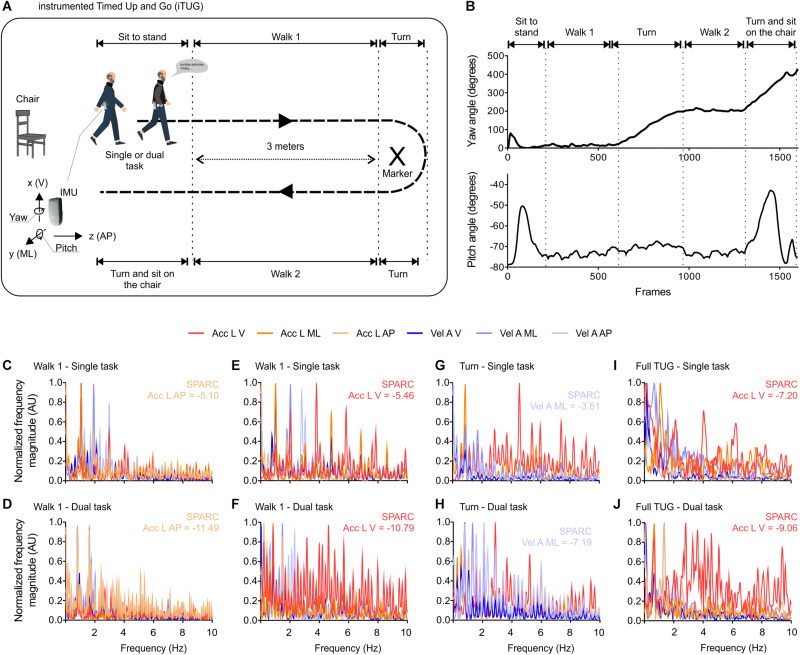
Instrumented timed up-and-go (iTUG) test and mobility smoothness (SPARC). **(A)** The TUG test was performed in single or dual task conditions, participants wore an inertial measurement unit (IMU) attached to the waist. The IMU measured linear accelerations (Acc L), angular velocities (Vel A), and angles in the three axes of movement. **(B)** TUG phases were identified using yaw and pitch angles. The full TUG and the following phases of the TUG were analyzed: sit-to-stand, walk 1, turn, walk 2, turn, and turn-to-sit (also depicted by arrows in **A**). **(C–J)** Representative spectral profiles used for the calculation of SPARC, note how some participants showed abundant frequency spectra above 5 Hz (expected to contain most of the frequencies components during steady-state walking). V: ventral; ML: mediolateral; AP: anteroposterior; SPARC: spectral arc length; Acc L: linear acceleration; Vel A: angular velocity; AU: adimensional unit; TUG: timed up-and-go; IMU: inertial measurement unit.

The iTUG was performed in different complexities, i.e., single or dual tasks. Angles extracted from the gyroscope were used to determine the duration of each TUG phase (Figure 1). The reliability of TUG phase detection using the above-mentioned procedure is shown in Supplementary Table S1. The analysis suggested an excellent between-assessor reliability in detecting most of the TUG phases (Cronbach’s α > 0.9). The turn and turn-to-sit phases obtained lower, but still acceptable, ICC (Cronbach’s α > 0.5). Fallers took longer to move from sit to the stand and turn to sit. Both groups took longer when performing the dual task and turning (see the statistics in Table 2). Although walking speed increased from the first to the third trial, movement smoothness (SPARC), overall, displayed the absence of trial effects.

**TABLE 2:** Duration and speed in the single and dual-task TUG tests.

	Single task—duration (s)				Dual task—duration (s)						
				
TUG phase	Fallers		Non-fallers		Fallers		Non-fallers			Main effects	
	Mean	SEM	Mean	SEM	Mean	SEM	Mean	SEM		*F*	*P*
Sit to stand—Trial 1	3.148	0.405	1.798	0.080	3.542	0.572	1.888	0.082	Group	**38.164**	**0.000**
Sit to stand—Trial 2	3.677	0.895	1.799	0.095	3.245	0.431	1.872	0.078	Trial	0.135	0.874
Sit to stand—Trial 3	3.220	0.600	1.753	0.088	3.154	0.500	1.855	0.096	Task	0.012	0.913
Walk 1—Trial 1	5.572	0.722	3.921	0.308	8.975	1.788	5.799	0.868	Group	**19.201**	**0.000**
Walk 1—Trial 2	5.500	0.919	3.736	0.365	8.387	1.595	5.343	0.470	Trial	0.851	0.428
Walk 1—Trial 3	5.430	0.809	3.468	0.300	7.552	1.531	4.237	0.433	Task	**13.851**	**0.000**
Turn—Trial 1	4.569	0.898	2.891	0.221	5.798	1.088	4.000	0.346	Group	**15.641**	**0.000**
Turn—Trial 2	4.327	0.654	2.973	0.299	5.240	0.925	3.806	0.267	Trial	1.049	0.351
Turn—Trial 3	3.723	0.417	2.940	0.325	4.736	0.763	3.373	0.239	Task	**6.767**	**0.010**
Walk 2—Trial 1	5.414	0.788	3.648	0.302	8.725	1.462	5.841	0.574	Group	**16.415**	**0.000**
Walk 2—Trial 2	5.140	0.736	3.604	0.419	7.452	1.098	5.400	0.587	Trial	2.070	0.128
Walk 2—Trial 3	4.758	0.649	3.412	0.373	6.276	1.192	4.559	0.595	Task	**19.369**	**0.000**
Turn to sit—Trial 1	4.323	0.496	2.976	0.178	4.895	0.820	3.623	0.269	Group	**24.548**	**0.000**
Turn to sit—Trial 2	4.412	0.730	2.820	0.266	4.529	0.561	3.317	0.219	Trial	0.766	0.466
Turn to sit—Trial 3	4.169	0.671	2.594	0.196	4.381	0.601	2.963	0.220	Task	2.019	0.156
Full TUG—Trial 1	23.026	3.099	15.233	0.971	31.935	5.391	21.151	1.886	Group	**24.193**	**0.000**
Full TUG—Trial 2	23.056	3.797	14.931	1.332	28.853	4.357	19.737	1.404	Trial	1.119	0.328
Full TUG—Trial 3	21.300	3.046	14.167	1.181	26.099	4.367	16.986	1.408	Task	**9.748**	**0.002**

	**Single task—speed (m.s^–1^)**				**Dual task—speed (m.s^–1^)**						
				
**TUG phase**	**Fallers**		**Non-fallers**		**Fallers**		**Non-fallers**			**Main effects**	
	**Mean**	**SEM**	**Mean**	**SEM**	**Mean**	**SEM**	**Mean**	**SEM**		***F***	***P***

Walk 1—Trial 1	0.717	0.058	0.888	0.057	0.553	0.057	0.669	0.047	Group	**24.120**	**0.000**
Walk 1—Trial 2	0.789	0.069	0.976	0.067	0.584	0.063	0.666	0.043	Trial	**4.135**	**0.017**
Walk 1—Trial 3	0.766	0.064	1.021	0.067	0.653	0.067	0.878	0.066	Task	**29.933**	**0.000**
Walk 2—Trial 1	0.773	0.060	0.951	0.061	0.547	0.060	0.639	0.047	Group	**19.157**	**0.000**
Walk 2—Trial 2	0.809	0.069	1.058	0.077	0.586	0.058	0.700	0.053	Trial	**6.583**	**0.002**
Walk 2—Trial 3	0.855	0.069	1.102	0.079	0.753	0.075	0.858	0.063	Task	**42.358**	**0.000**

*Three-way ordinary ANOVA (df = 372) with factors group (fallers X non-fallers; df = 1). trial (1 × 2 × 3; df = 2) and task (single X dual; df = 1). All interactions were not significant (not shown). Bold values denote statistical significance (*p* < 0.05).*

### Mobility Smoothness Between Fallers and Non-fallers: Walking Bouts

**FIGURE 2:**
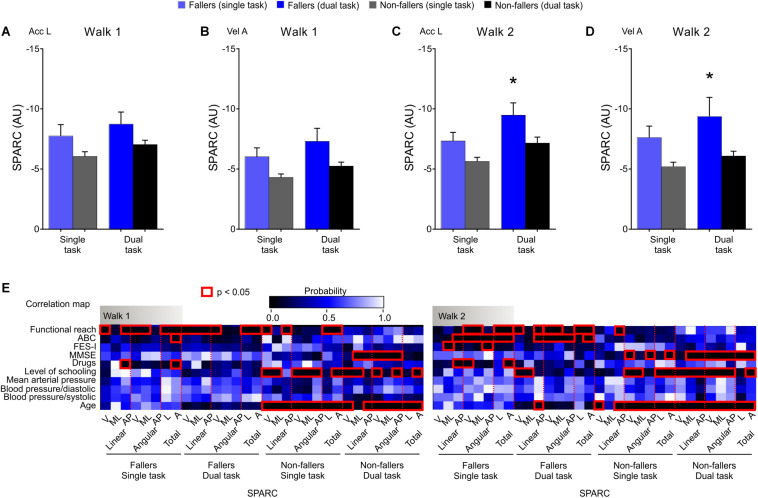
Walking smoothness (SPARC) outcomes were stable across trials and reduced for fallers. **(A–D)** SPARC outcomes during walk 1 and 2 showed group and task, but not trial, main effects, thus we collapsed the three trials. Group and time effects were evident (Table 3), alongside with *post hoc* effect for the dual task walk 2 phase. **(E)** The correlation map analysis showed a consistent correlation between functional reach, ABC scale, and movement smoothness in both the fallers and non-fallers. Correlations were also found with MMSE, level of schooling, and age for non-fallers. Two-way repeated-measures ANOVA with factors time (single X dual task) and group (fallers X non-fallers) followed by Sidak-correction (*post hoc*). Data are shown as mean ± SEM; *n*_Fallers_ = 32; *n*_Non–fallers_ = 32; *p < 0.05 (*post hoc*); red rectangles in **E** are *p < 0.05 (Spearman correlation). V: ventral; ML: mediolateral; AP: anteroposterior; SPARC: spectral arc length; Acc L: linear acceleration; Vel A: angular velocity; AU: adimensional unit; TUG: timed up-and-go.

Given the absence of trial effects, we averaged the SPARC data from all trials to express the participant’s performance (Table 3). SPARC was lower for fallers, displayed task effects, and absence of trial effects (except for turn Acc L—dual task; explored below). SPARC metrics also showed several correlations with the functional reach and ABC score for fallers. Additionally, mobility smoothness was also correlated with other variables, such as MMSE, level of schooling, and age for non-fallers (Figure 2 and Supplementary Table S3).

**TABLE 3:** Main effects of time and group for SPARC Acc L total and SPARC Vel A total.

	SPARC Acc L total		SPARC Vel A total	
TUG phase	Time	Group	Time	Group
Sit to stand	***F*(1,62) = 27.19; *p* < 0.0001**	***F*(1,62) = 4.65; *p* = 0.0350**	***F*(1,62) = 24.24; *p* < 0.0001**	***F*(1,62) = 4.20; *p* = 0.0447**
Walk 1	***F*(1,62) = 14.68; *p* = 0.0003**	*F*(1,62) = 2.90; *p* = 0.0939	***F*(1,62) = 18.76; *p* < 0.0001**	***F*(1,62) = 4.01; *p* = 0.0497**
Turn	***F*(1,62) = 66.49; *p* < 0.0001**	*F*(1,62) = 3.30; *p* = 0.0739	***F*(1,62) = 47.77; *p* < 0.0001**	***F*(1,62) = 4.55; *p* = 0.0369**
Walk 2	***F*(1,62) = 58.99; *p* < 0.0001**	***F*(1,62) = 4.47; *p* = 0.0385**	***F*(1,62) = 12.69; *p* = 0.0007**	***F*(1,62) = 4.79; *p* = 0.0323**
Turn to sit	*F*(1,62) = 3.06; *p* = 0.0852	***F*(1,62) = 8.33; *p* = 0.0054**	*F*(1,62) = 0.09; *p* = 0.7633	***F*(1,62) = 7.98; *p* = 0.0064**
Full TUG	*F*(1, 62) = 0.76; *p* = 0.3867	***F*(1,62) = 7.31; *p* = 0.0088**	***F*(1,62) = 7.58; *p* = 0.0077**	***F*(1,62) = 6.18; *p* = 0.0156**

*Two-way repeated-measures ANOVA with factors time (single X dual task) and group (fallers X non-fallers). All interactions were not significant (not shown). Bold values denote statistical significance (*p* < 0.05).*

### Mobility Smoothness Between Fallers and Non-fallers: Turn and Full TUG

**FIGURE 3:**
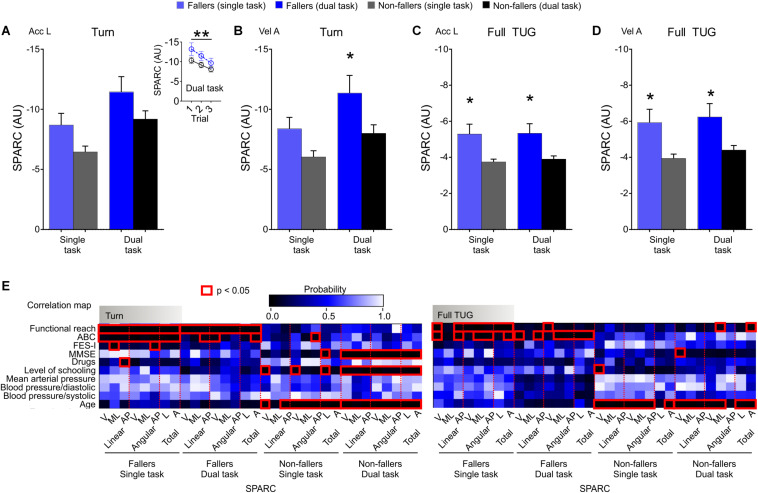
Turning displayed drastically reduced smoothness (SPARC) values for oldest-old fallers and non-fallers. **(A)** The turning phase of the TUG test showed reduced smoothness (SPARC Acc L) mostly in the first trial, but increased movement smoothness was evident between trials (inset). The between-trial collapsed data showed the absence of group effects. **(B)** The SPARC angular component (SPARC Vel A) during turning, on the other hand, displayed group effects and a *post hoc* effect for the dual task. **(C,D)** During the full TUG, the SPARC was reduced for fallers performing single or dual tasks. **(E)** Similar to the walking bouts, SPARC metrics during turning consistently correlated with the functional reach test and ABC scale for fallers. Non-fallers also showed correlations with MMSE, level of schooling, and age. Note that when the full TUG is considered, non-fallers showed fewer correlations with the MMSE and level of schooling. Two-way repeated-measures ANOVA with factors time (single X dual task) and group (fallers X non-fallers) followed by Sidak correction (*post hoc*). Data are mean ± SEM; *n*_Fallers_ = 32; *n*_Non–fallers_ = 32; *p < 0.05 (*post hoc*); **p < 0.05 trial effect in the ordinary ANOVA (group Fallers X Non-fallers; trial 1 × 2 × 3; task Single X Dual); red rectangles in **E** are *p < 0.05 (Spearman correlation). V: ventral; ML: mediolateral; AP: anteroposterior; SPARC: spectral arc length; Acc L: linear acceleration; Vel A: angular velocity; AU: adimensional unit; TUG: timed up-and-go.

In addition to steady-state walking, we analyzed other aspects of functional mobility such as turning while walking. Indeed, fallers exhibited a notable reduction in movement smoothness during the turn phase of the TUG. While there was no between-trial learning effect for the time taken to turn (i.e., time to turn was stable between trials), SPARC showed a change from trial 1 to 3 during the turn phase of the TUG (Figure 3). Together, these findings suggest the participants experienced difficulty maintaining good levels of mobility smoothness when performing faster movements in less stable conditions, as typically occur during the turning phase of the TUG. In agreement with walk 1 and 2 phases of the TUG, SPARC metrics during the turning phase were consistently correlated with the functional reach and ABC score for fallers; but also correlated with MMSE, level of schooling, and age for non-fallers (particularly during the dual task; Figure 3). Altogether, these findings indicate the oldest-old have a pronounced difficulty turning smoothly and, subsequently, an adaptive motor control change is required to complete the task, which is not possible to assess using the traditional TUG with a stopwatch.

SPARC metrics displayed stronger group differences (ES ≈ 4.18) compared to the time taken to complete the full TUG test (ES = 2.96) (Figures 2, 3). Again, consistent correlations between SPARC in the full TUG, the functional reach and ABC score for fallers were found. Movement smoothness of the participants without a history of falls was only correlated with age when considering the full TUG test (Figure 3).

Fallers showed reduced smoothness (SPARC) while transitioning from “sit to stand” during the dual task. The final phase “turn to sit” was not affected by the task. This is expected since most participants stopped or reduced the vocalization of the weekdays in this phase of the dual task TUG (Supplementary Figure S3).

## Discussion

Here we described mobility smoothness during the TUG, a widely used test (Barry et al., 2014), applying robust and sensitive movement smoothness metrics (SPARC). This study supports the iTUG test in providing a smoothness mobility index, which may contribute to understanding functional mobility and falls in community-dwelling oldest-old. Fallers took longer to complete the test and exhibited reduced smoothness when performing the single and dual task TUG. In comparison to the traditional TUG test outcome (duration), mobility smoothness may reveal larger between-group effect sizes between oldest-old with and without a history of falls. Moreover, this is the first study to highlight how the iTUG can provide valuable insights into the relation between mobility smoothness and time taken to move when performing different phases of a walking-based task. For instance, the time taken to complete the sit-to-stand phase of the TUG is different between fallers and non-fallers, whereas mobility smoothness showed no *post hoc* effect. Interestingly, during the turning and standing to sit phases, fallers and non-fallers did not differ regarding the time spent to complete these phases of the test, but mobility smoothness was drastically affected for participants with a history of falls. The movement smoothness also showed remarkable positive correlations with the ability to maintain stability during the functional reach test in the group of fallers. Additionally, there was a consistent correlation between SPARC metrics and age and less consistent relation with MMSE and the level of schooling in the non-fallers.

Dual task situations involve attention and other cognitive processes of importance to posture and locomotion (Bayot et al., 2018). However, the complexity of the TUG test was previously shown to add little value when assessing falls in older adults (age ≈ 76 years) (Asai et al., 2018). Here, we reported similar findings for mobility smoothness as both fallers and non-fallers displayed reduced movement smoothness while performing the dual task TUG. Movement smoothness was noticeably low during turning in the dual task TUG, ranging up to -13 for fallers, a higher value when compared with previous reports of -5.5 for walking (Beck et al., 2018) or -6 for turning (Parkinson’s disease; Pinto et al., 2019). Difficulties of turning while walking in the oldest-old are also evident in the adaptation pattern of mobility smoothness from the first to the third trial of the dual task. This may indicate the oldest-old adapt motor learning and behavior to overcome the difficulties experienced when turning while walking, for example, by using different movement strategies (Weiss et al., 2016). Interestingly, this adaptation was not noticeable in the traditional TUG duration outcomes, which were stable between trials. Turning involves approximately 40% of all the steps taken during the activities of daily living, depending on how often certain activities are performed (Glaister et al., 2007), and sharp turns, such as the 180° turns during the TUG test, are energetically demanding (Justine et al., 2014). Altogether, these findings suggest the turning phase is a challenging situation for the oldest-old at risk of falls. This is in line with research showing older adults and stroke survivors have similar deficits when turning 90° under dual task conditions (Hollands et al., 2014). Together, the current findings contribute to consolidating the importance of turning assessment as a marker to explain falls in the oldest-old.

Similarly, standing up from a chair is a dynamic equilibrium task susceptible to age-related modifications (Mourey et al., 2000). When evaluating movement smoothness and time taken to transit from sit to stand together, we found an interesting behavior in fallers (Supplementary Figure S1). The cautious transition of fallers during the sit to stand phase may represent an attempt to control horizontal motion and increase movement smoothness to avoid falls (Mourey et al., 2000). By contrast, while turning and transitioning from turn to sit, there was little difference between fallers and non-fallers in terms of time taken, but mobility smoothness was markedly different (ES ≈ 4.59). These findings highlight the importance of evaluating mobility smoothness in addition to movement duration in the TUG test, with important implications for fall assessment.

Day-to-day gait speed and variability involve several factors and may be linked to different brain networks in vulnerable older adults (Lo et al., 2017). Falls risk may be missed if walking speed exceeds a threshold on a given day or a given trial after learning/performance effects on the TUG are seen. Our results support the concept of SPARC as a stable, robust, and sensitive measurement of movement smoothness regardless of movement speed and duration (Balasubramanian et al., 2012; Balasubramanian, 2015) likely able to identify falls risk regardless of speed ability on the day.

Indeed, increased speed of walking bouts between trials is not reflected in the SPARC outcomes. In addition, SPARC is ≈10 times less susceptible to signal-to-noise ratio artifacts (Balasubramanian et al., 2012; Balasubramanian, 2015), a particularly important point when analyzing accelerometric data, which are more prone to noise than kinematic data, for example. Furthermore, the mobility smoothness data from SPARC metrics in the TUG provide new insights into mobility, especially when considered concerning duration and speed, with a noteworthy power to distinguish between fallers and non-fallers. Altogether, the findings described here support the use of SPARC during the TUG test to evaluate age-related motor control decline and its association with a history of falls.

In addition, correlation maps suggest that further studies should address the association between mobility smoothness, a history of falls, and unhealthy habits such as physical inactivity and subsequent depressive symptoms (Jorgensen et al., 2002; Hollands et al., 2010; Schuch et al., 2016).

Finally, current data support using SPARC metrics in the iTUG is feasible and allow the test conduction outside of the lab, which might provide realistic data, since laboratory kinematic measurements (e.g., axial segment coordination) have failed in distinguishing a history of falls in stroke survivors (Hollands et al., 2010).

This study has some limitations. The convenience sample may have influenced the effect sizes of the SPARC metrics. While the process of determining the duration of each TUG test phase has been reliably conducted by the assessors in this research group, visual inspection of the signal may prove challenging for untrained assessors. Moreover, the ICC results suggest the SPARC outcomes are reliable, with the exception of the final TUG phase (turn and sit on the chair). This is probably due to the wide range of motor strategies adopted by the oldest-old during this TUG phase. For example, some participants stopped during the final part of the turn and, after a pause, sat. Others performed a combined turning and sitting movement or turned completely before sitting without a between-subphase pause. Overall, in the oldest-old, the signals from this TUG phase are challenging, even for trained experts, and are a matter for further research. Thus, the SPARC results in the “turn to sit on the chair” phase of the TUG should be interpreted with caution. Further studies should compare data from the G-Walk software, manual detection (as performed here), and kinematics as the gold standard. In addition, while the retrospective analysis revealed SPARC is associated with history of falls, we do not know the extent to which it can be used to independently predict future falls. Thus, more research is necessary to assess the value of movement smoothness as a predictor of the likelihood of future falls.

## Conclusion

This study characterized mobility smoothness in elderly subjects aged 85 and older during a functional mobility task involving different degrees of complexity. Using robust SPARC smoothness metrics, we identified important factors associated with a history of falls, namely, age, unsmooth movements, and performance in a functional reach task. We suggest including mobility smoothness measures in the iTUG to ensure movement quality is more fully assessed in the test. These findings provide a better understanding of functional mobility in the oldest-old and may be of importance when assessing the efficacy of fall prevention programs.

## Data Availability Statement

The datasets generated for this study are available on request to the corresponding author.

## Ethics Statement

This study protocol was reviewed and approved by the Research Ethics Committee of the Pontifical Catholic University of Rio Grande do Sul. The participants provided their written informed consent to participate in this study.

## Author Contributions

AF carried out the data collection, conceived and planned the experiments, analyzed the data, and contributed to the writing of the manuscript. GB designed the model framework, performed signal analyses, analyzed the data, and contributed to the writing of the manuscript. FB and AS carried out the data collection and reviewed the manuscript. RB contributed to implementation of the research and reviewed the manuscript. AP and KH conceived of the study design and reviewed the manuscript. RM conceived of the presented idea, supervised the findings, and wrote and reviewed the manuscript. All authors discussed the results and contributed to the final manuscript.

## Conflict of Interest

The authors declare that the research was conducted in the absence of any commercial or financial relationships that could be construed as a potential conflict of interest.

## Supplementary Material

FIGURE S1 Data analysis to extract the TUG task phases. The pitch **(a)** and yaw **(b)** angles were extracted from the IMU. **(a)** The pitch angle was used to detect the trunk flexion and extension, characteristic of the sit and the stand movements. These movements are depicted between the vertical lines 1a and 2a (sit to stand), and 3a and 4a (turn to sit), the trunk flexion is a decrease in the pitch angle and the trunk extension is an increase in the pitch angle. **(b)** Next, the yaw angle was used to detect the walk and the turn phases. Importantly, the yaw angle is also used to fine-tune the detection of the turn and sit on the chair phase (vertical line 3a in **a**) and to assist in the detection of the walk 1 phase. The walk 1 start frame (vertical line 1b) is set at the same index of the vertical line 2a (≈200 frames in this example). The end of the walk 1 phase and the start of the turn phase is detected when the yaw angle starts to change (2b), the turn is considered from this point until a 180° change in the yaw angle occurred. This 180° turn is followed by another period of oscillatory yaw angle variation (from 3b to 4b; walk 2 phase). At this point in the visual inspection procedure, the evaluator searched for the final turn, which occurs before sitting and used this value (4b) to fine-tune the detection of the turn and sit on the chair phase (≈1100 frames in this example; 3a in **a**).

FIGURE S2 Statistical plan. This step-by-step plan illustrates the statistical choices and decisions.

FIGURE S3 Fallers displayed reduced smoothness while standing from a chair or turning and sitting on the chair, but the latter displayed absence of task effect. **(a,b)** Fallers showed reduced smoothness (SPARC) while transitioning from the sitting to the standing position during the dual task. **(c,d)** The final TUG transition “turn-to-sit” was not affected by the task. **(e)** The representative spectral profile of a participant performing the “turn-to-sit” phase of the TUG task under single and dual task conditions. Note the mild SPARC values and the lack of noticeable difference between single and dual task. V: ventral; ML: mediolateral; AP: anteroposterior; SPARC: spectral arc length; Acc L: linear acceleration; Vel A: angular velocity; AU: adimensional unit; TUG: time up-and-go.

TABLE S1 Reliability statistics: Two independent assessors determined the TUG phases detection in a random sub-sample (24 participants).

TABLE S2 Three-way ordinary ANOVA summary. This was used as a first step to explore the dataset.

TABLE S3 Significant correlations between SPARC, demographics, and functional variables.
