# Supplementary figures and images for: SPARC Metrics Provide Mobility Smoothness Assessment in Oldest-Old With and Without a History of Falls: A Case Control Study
Source: Front Physiol. 2020 Jun 10;11:540. doi: 10.3389/fphys.2020.00540 (PMC7298141; doi:10.3389/fphys.2020.00540)

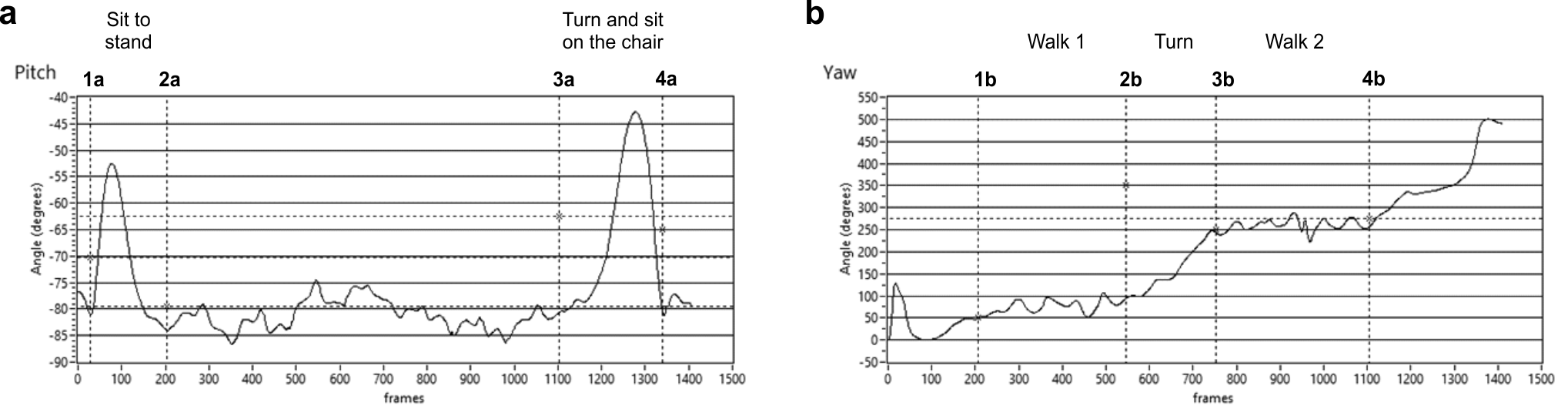

Supplement: FIGURE S1 — Data analysis to extract the TUG task phases. The pitch (a) and yaw (b) angles were extracted from the IMU. (a) The pitch angle was used to detect the trunk flexion and extension, characteristic of the sit and the stand movements. These movements are depicted between the vertical lines 1a and 2a (sit to stand), and 3a and 4a (turn to sit), the trunk flexion is a decrease in the pitch angle and the trunk extension is an increase in the pitch angle. (b) Next, the yaw angle was used to detect the walk and the turn phases. Importantly, the yaw angle is also used to fine-tune the detection of the turn and sit on the chair phase (vertical line 3a in a) and to assist in the detection of the walk 1 phase. The walk 1 start frame (vertical line 1b) is set at the same index of the vertical line 2a (≈200 frames in this example). The end of the walk 1 phase and the start of the turn phase is detected when the yaw angle starts to change (2b), the turn is considered from this point until a 180° change in the yaw angle occurred. This 180° turn is followed by another period of oscillatory yaw angle variation (from 3b to 4b; walk 2 phase). At this point in the visual inspection procedure, the evaluator searched for the final turn, which occurs before sitting and used this value (4b) to fine-tune the detection of the turn and sit on the chair phase (≈1100 frames in this example; 3a in a). [file Image_1.TIF]

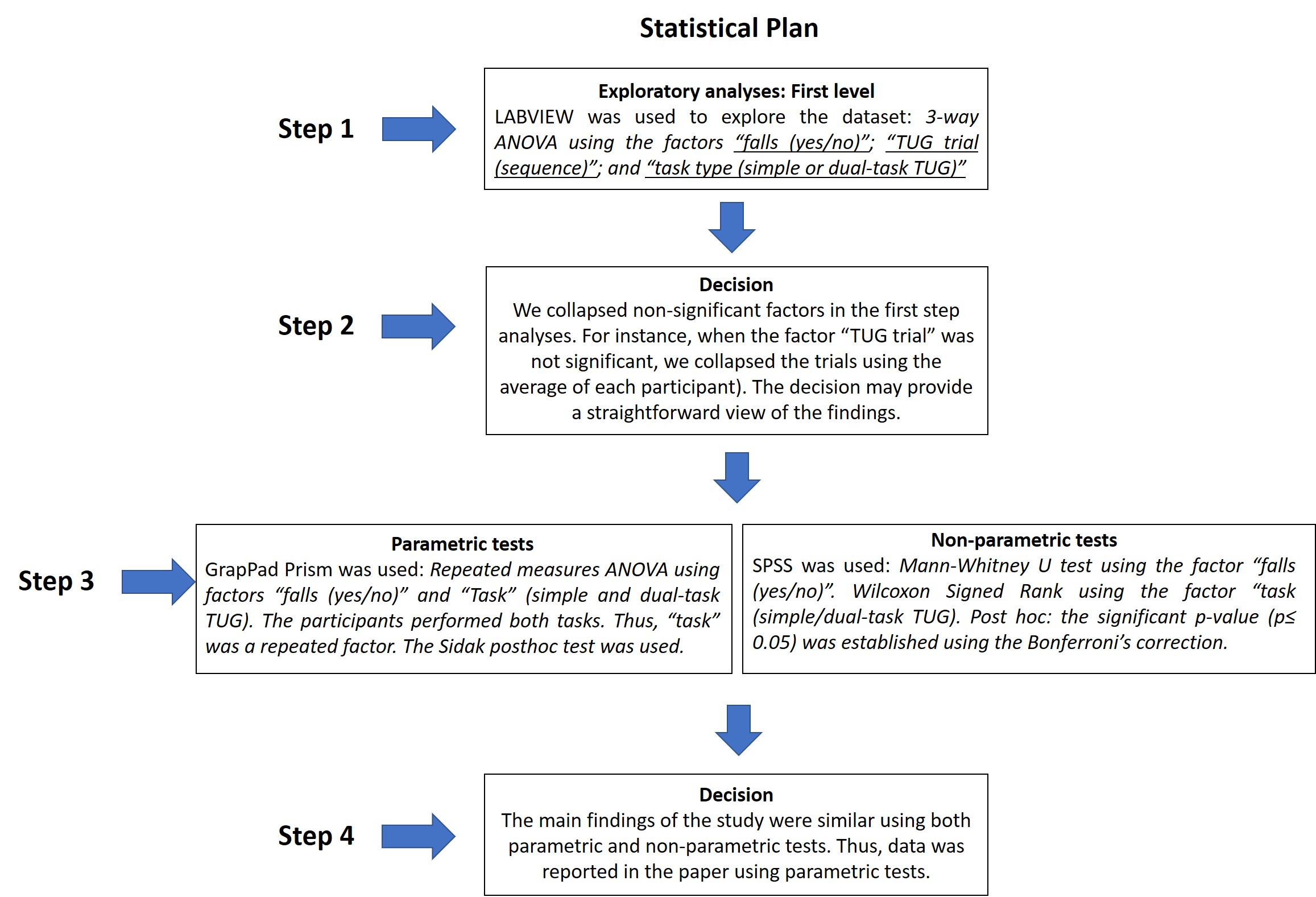

Supplement: FIGURE S2 — Statistical plan. This step-by-step plan illustrates the statistical choices and decisions. [file Image_2.JPEG]

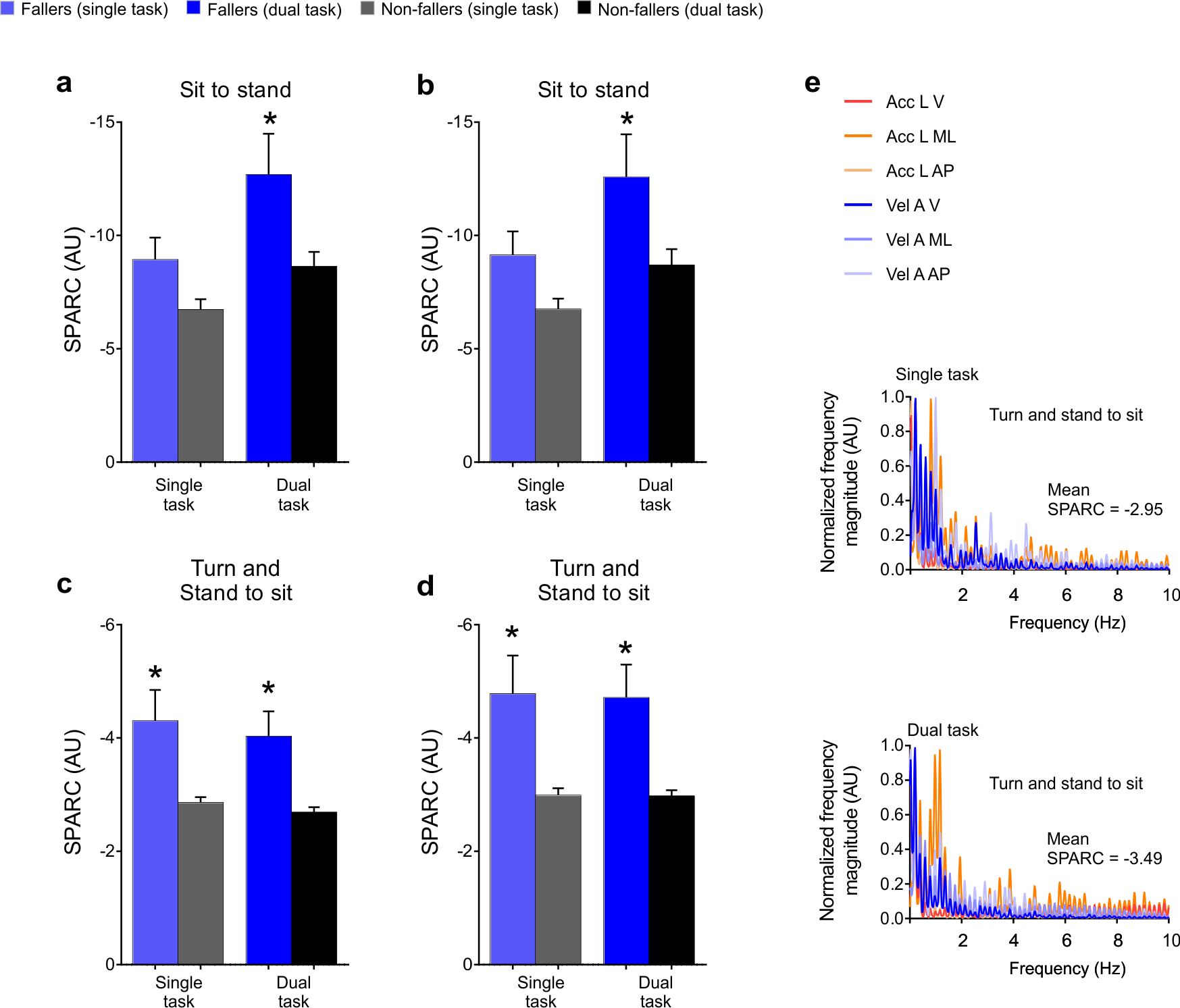

Supplement: FIGURE S3 — Fallers displayed reduced smoothness while standing from a chair or turning and sitting on the chair, but the latter displayed absence of task effect. (a,b) Fallers showed reduced smoothness (SPARC) while transitioning from the sitting to the standing position during the dual task. (c,d) The final TUG transition “turn-to-sit” was not affected by the task. (e) The representative spectral profile of a participant performing the “turn-to-sit” phase of the TUG task under single and dual task conditions. Note the mild SPARC values and the lack of noticeable difference between single and dual task. V: ventral; ML: mediolateral; AP: anteroposterior; SPARC: spectral arc length; Acc L: linear acceleration; Vel A: angular velocity; AU: adimensional unit; TUG: time up-and-go. [file Image_3.TIF]
