# Supplementary material for: SPARC Metrics Provide Mobility Smoothness Assessment in Oldest-Old With and Without a History of Falls: A Case Control Study
Source: Front Physiol. 2020 Jun 10;11:540. doi: 10.3389/fphys.2020.00540 (PMC7298141; doi:10.3389/fphys.2020.00540)
Supplement: TABLE S1 — Reliability statistics: Two independent assessors determined the TUG phases detection in a random sub-sample (24 participants). [file Table_1.DOCX]

| **Supplementary Table 1.** Reliability Statistics: Two independent assessors determined the TUG phases detection in a random sub-sample (24 participants). | | | | | | | | | | | | | | | | |
| --- | --- | --- | --- | --- | --- | --- | --- | --- | --- | --- | --- | --- | --- | --- | --- | --- |
|  |  |  |  |  |  |  |  |  |  | **Reliability** | | | | | | |
| **Variable** |  | **Assessor 1** | | |  | **Assessor 2** | | |  |  |  | **Intraclass correlation coefficient** | | | | |
| **Speed and duration** |  | **Mean** |  | **SD** |  | **Mean** |  | **SD** |  | **Cronbach's Alpha** |  | **Intraclass correlation** |  | **Lower bound** |  | **Upper bound** |
| Walk 1 speed (m.s^-1^) |  | 0.690 |  | 0.363 |  | 0.719 |  | 0.331 |  | 0.971 |  | 0.971* |  | 0.933 |  | 0.988 |
| Walk 2 speed (m.s^-1^) |  | 0.830 |  | 0.371 |  | 0.842 |  | 0.402 |  | 0.948 |  | 0.948* |  | 0.880 |  | 0.978 |
| Walk 1 duration (s) |  | 5.851 |  | 3.714 |  | 5.519 |  | 3.479 |  | 0.980 |  | 0.980* |  | 0.955 |  | 0.992 |
| Turn duration (s) |  | 3.276 |  | 1.609 |  | 3.690 |  | 1.793 |  | 0.929 |  | 0.929* |  | 0.835 |  | 0.969 |
| Walk 2 duration (s) |  | 4.990 |  | 3.631 |  | 4.910 |  | 3.467 |  | 0.970 |  | 0.970* |  | 0.932 |  | 0.987 |
| Sit to stand duration (s) |  | 2.121 |  | 0.957 |  | 2.205 |  | 1.064 |  | 0.977 |  | 0.977* |  | 0.947 |  | 0.990 |
| Turn to sit duration (s) |  | 2.766 |  | 1.214 |  | 3.586 |  | 2.199 |  | 0.676 |  | 0.676* |  | 0.252 |  | 0.860 |
| Full TUG duration (s) |  | 19.005 |  | 9.376 |  | 19.909 |  | 10.744 |  | 0.990 |  | 0.990* |  | 0.976 |  | 0.995 |
| **SPARC (Acc L)** |  | **Mean** |  | **SD** |  | **Mean** |  | **SD** |  | **Cronbach's Alpha** |  | **Intraclass correlation** |  | **Lower bound** |  | **Upper bound** |
| Walk 1 - SPARC Acc L |  | -9.584 |  | 4.966 |  | -9.142 |  | 5.122 |  | 0.969 |  | 0.969* |  | 0.927 |  | 0.986 |
| Turn - SPARC Acc L |  | -6.009 |  | 2.309 |  | -6.849 |  | 2.564 |  | 0.792 |  | 0.792* |  | 0.519 |  | 0.910 |
| Walk 2 - SPARC Acc L |  | -8.136 |  | 4.615 |  | -8.283 |  | 4.111 |  | 0.946 |  | 0.946* |  | 0.876 |  | 0.977 |
| Sit to stand - SPARC Acc L |  | -3.033 |  | 1.066 |  | -3.057 |  | 1.004 |  | 0.877 |  | 0.877* |  | 0.715 |  | 0.947 |
| Turn to sit - SPARC Acc L |  | -3.794 |  | 1.094 |  | -4.630 |  | 2.570 |  | 0.436 |  | 0.436 |  | -0.305 |  | 0.756 |
| Full TUG - SPARC Acc L |  | -6.364 |  | 2.820 |  | -6.572 |  | 3.420 |  | 0.970 |  | 0.970* |  | 0.931 |  | 0.987 |
|  |  |  |  |  |  |  |  |  |  |  |  |  |  |  |  |  |
| **SPARC (Vel A)** |  | **Mean** |  | **SD** |  | **Mean** |  | **SD** |  | **Cronbach's Alpha** |  | **Intraclass correlation** |  | **Lower bound** |  | **Upper bound** |
| Walk 1 - SPARC Vel A |  | -9.444 |  | 4.759 |  | -9.131 |  | 4.882 |  | 0.957 |  | 0.957* |  | 0.902 |  | 0.982 |
| Turn - SPARC Vel A |  | -4.977 |  | 2.426 |  | -5.374 |  | 2.047 |  | 0.841 |  | 0.841* |  | 0.634 |  | 0.931 |
| Walk 2 - SPARC Vel A |  | -8.174 |  | 4.997 |  | -8.276 |  | 4.764 |  | 0.970 |  | 0.970* |  | 0.930 |  | 0.987 |
| Sit to stand - SPARC Vel A |  | -3.200 |  | 1.207 |  | -3.321 |  | 1.094 |  | 0.933 |  | 0.933* |  | 0.845 |  | 0.971 |
| Turn to sit - SPARC Vel A |  | -4.192 |  | 1.590 |  | -5.308 |  | 2.681 |  | 0.701 |  | 0.701* |  | 0.309 |  | 0.871 |
| Full TUG - SPARC Vel A |  | -6.867 |  | 3.243 |  | -6.903 |  | 3.513 |  | 0.993 |  | 0.993* |  | 0.983 |  | 0.997 |
|  |  |  |  |  |  |  |  |  |  |  |  |  |  |  |  |  |
| *p < 0.05. Two-way mixed-effects model where people effects are random and measures effects are fixed. This estimate is computed assuming the interaction effect is absent. because it is not estimable otherwise. | | | | | | | | | | | | | | | | |
