# Supplementary material for: SPARC Metrics Provide Mobility Smoothness Assessment in Oldest-Old With and Without a History of Falls: A Case Control Study
Source: Front Physiol. 2020 Jun 10;11:540. doi: 10.3389/fphys.2020.00540 (PMC7298141; doi:10.3389/fphys.2020.00540)
Supplement: TABLE S2 — Three-way ordinary ANOVA summary. This was used as a first step to explore the dataset. [file Table_2.DOCX]

| **Supplementary Table 2.** 3-way ordinary ANOVA summary. This was used as a first step to explore the dataset. | | | | | | | | |
| --- | --- | --- | --- | --- | --- | --- | --- | --- |
|  |  | **Factors** | | | **Interactions** | | | |
|  |  | Falls | Trial | Task | Falls x Trial | Falls x Task | Trial x Task | Falls x Trial x Task |
| Walk 1 speed (m.s^-1^) | F | **24.120** | **4.135** | **29.933** | 0.928 | 0.832 | 1.125 | 0.099 |
|  | p | **0.000** | **0.017** | **0.000** | 0.396 | 0.362 | 0.326 | 0.906 |
| Walk 2 speed (m.s^-1^) | F | **19.157** | **6.583** | **42.358** | 0.149 | 2.576 | 0.923 | 0.053 |
|  | p | **0.000** | **0.002** | **0.000** | 0.861 | 0.109 | 0.398 | 0.948 |
| Walk 1 duration (s) | F | **19.201** | 0.851 | **13.851** | 0.018 | 1.492 | 0.384 | 0.004 |
|  | p | **0.000** | 0.428 | **0.000** | 0.982 | 0.223 | 0.681 | 0.996 |
| Turn duration (s) | F | **15.641** | 1.049 | **6.767** | 0.294 | 0.134 | 0.136 | 0.051 |
|  | p | **0.000** | 0.351 | **0.010** | 0.746 | 0.714 | 0.873 | 0.950 |
| Walk 2 duration (s) | F | **16.415** | 2.070 | **19.369** | 0.252 | 0.517 | 0.777 | 0.061 |
|  | p | **0.000** | 0.128 | **0.000** | 0.777 | 0.472 | 0.461 | 0.941 |
| Sit to stand duration (s) | F | **38.164** | 0.135 | 0.012 | 0.083 | 0.063 | 0.251 | 0.232 |
|  | p | **0.000** | 0.874 | 0.913 | 0.920 | 0.801 | 0.778 | 0.793 |
| Turn and stand to sit duration (s) | F | **24.548** | 0.766 | 2.019 | 0.036 | 0.130 | 0.135 | 0.026 |
|  | p | **0.000** | 0.466 | 0.156 | 0.964 | 0.718 | 0.874 | 0.974 |
| Total duration (s) | F | **24.193** | 1.119 | **9.748** | 0.037 | 0.317 | 0.351 | 0.027 |
|  | p | **0.000** | 0.328 | **0.002** | 0.964 | 0.574 | 0.704 | 0.974 |
| SPARC Acc L total - Sit to stand | F | **22.328** | 0.930 | **18.192** | 0.103 | 1.898 | 0.538 | 0.004 |
|  | p | **0.000** | 0.395 | **0.000** | 0.903 | 0.169 | 0.584 | 0.996 |
| SPARC Vel A total - Sit to stand | F | **20.661** | 0.871 | **15.163** | 0.015 | 1.159 | 0.363 | 0.014 |
|  | p | **0.000** | 0.420 | **0.000** | 0.985 | 0.282 | 0.696 | 0.986 |
| SPARC Acc L total - Walk 1 | F | **14.345** | 0.734 | **4.746** | 0.085 | 0.000 | 0.115 | 0.140 |
|  | p | **0.000** | 0.481 | **0.030** | 0.919 | 0.997 | 0.891 | 0.869 |
| SPARC Vel A total - Walk 1 | F | **20.730** | 0.734 | **7.114** | 0.403 | 0.170 | 0.037 | 0.036 |
|  | p | **0.000** | 0.481 | **0.008** | 0.669 | 0.680 | 0.963 | 0.964 |
| SPARC Acc L total - Turn | F | **16.760** | **3.359** | **24.917** | 0.224 | 0.001 | 1.504 | 0.054 |
|  | p | **0.000** | **0.036** | **0.000** | 0.799 | 0.978 | 0.224 | 0.948 |
| SPARC Vel A total - Turn | F | **23.119** | 2.261 | **17.349** | 0.278 | 0.728 | 0.674 | 0.144 |
|  | p | **0.000** | 0.106 | **0.000** | 0.758 | 0.394 | 0.510 | 0.866 |
| SPARC Acc L total - Walk 2 | F | **22.037** | 0.629 | **18.427** | 0.238 | 0.524 | 1.186 | 0.224 |
|  | p | **0.000** | 0.533 | **0.000** | 0.788 | 0.470 | 0.306 | 0.799 |
| SPARC Vel A total -Walk 2 | F | **25.358** | 0.489 | **5.368** | 0.128 | 0.599 | 0.043 | 0.060 |
|  | p | **0.000** | 0.614 | **0.021** | 0.880 | 0.439 | 0.958 | 0.942 |
| SPARC Acc L total - Turn and stand to sit | F | **39.247** | 0.197 | 1.009 | 0.158 | 0.053 | 0.367 | 0.346 |
|  | p | **0.000** | 0.822 | 0.316 | 0.854 | 0.819 | 0.693 | 0.708 |
| SPARC Vel A total - Turn and stand to sit | F | **38.505** | 0.204 | 0.020 | 0.178 | 0.009 | 0.079 | 0.082 |
|  | p | **0.000** | 0.816 | 0.887 | 0.837 | 0.925 | 0.924 | 0.922 |
| SPARC Acc L total - Full TUG | F | **36.676** | 1.423 | 0.152 | 0.086 | 0.056 | 0.201 | 0.168 |
|  | p | **0.000** | 0.242 | 0.696 | 0.917 | 0.813 | 0.818 | 0.845 |
| SPARC Vel A total - Full TUG | F | **33.133** | 0.708 | 1.347 | 0.037 | 0.058 | 0.129 | 0.012 |
|  | P | **0.000** | 0.493 | 0.246 | 0.964 | 0.809 | 0.879 | 0.988 |
| Significant main factors are in bold (with a light grey background). Exploratory 3-way ordinary ANOVA. p < 0.05. | | | | | | | | |
