# Supplementary material for: SPARC Metrics Provide Mobility Smoothness Assessment in Oldest-Old With and Without a History of Falls: A Case Control Study
Source: Front Physiol. 2020 Jun 10;11:540. doi: 10.3389/fphys.2020.00540 (PMC7298141; doi:10.3389/fphys.2020.00540)
Supplement: TABLE S3 — Significant correlations between SPARC, demographics, and functional variables. [file Table_3.DOCX]

| **Supplementary Table 3.** Significant correlations between SPARC, demographics and functional variables. | | | | | | | | | | | | | | | |
| --- | --- | --- | --- | --- | --- | --- | --- | --- | --- | --- | --- | --- | --- | --- | --- |
|  |  |  |  |  |  |  |  |  |  |  |  |  |  |  |  |
| Sit to stand | Fallers (single task) |  | |  | Fallers (dual task) |  | |  | Non-fallers (single task) |  | |  | Non-fallers (dual task) |  | |
|  |  | ρ | p |  |  | ρ | p |  |  | ρ | p |  |  | ρ | p |
|  | ABC*SPARC Acc A ML | 0.439 | 0.012 |  | ABC*SPARC Acc A ML | 0.471 | 0.007 |  | Age*SPARC Acc A AP | -0.516 | 0.003 |  | Age*SPARC Acc A AP | -0.498 | 0.004 |
|  | ABC*SPARC Acc A Total | 0.387 | 0.029 |  | ABC*SPARC Acc A Total | 0.430 | 0.014 |  | Age*SPARC Acc A ML | -0.383 | 0.031 |  | Age*SPARC Acc A ML | -0.478 | 0.006 |
|  | ABC*SPARC Acc A V | 0.455 | 0.009 |  | ABC*SPARC Acc A V | 0.436 | 0.013 |  | Age*SPARC Acc A Total | -0.578 | 0.001 |  | Age*SPARC Acc A Total | -0.527 | 0.002 |
|  | ABC*SPARC Acc L AP | 0.359 | 0.044 |  | ABC*SPARC Acc L AP | 0.445 | 0.011 |  | Age*SPARC Acc A V | -0.623 | 0.000 |  | Age*SPARC Acc A V | -0.522 | 0.002 |
|  | ABC*SPARC Acc L Total | 0.437 | 0.012 |  | ABC*SPARC Acc L ML | 0.440 | 0.012 |  | Age*SPARC Acc L AP | -0.443 | 0.011 |  | Age*SPARC Acc L AP | -0.578 | 0.001 |
|  | ABC*SPARC Acc L V | 0.360 | 0.043 |  | ABC*SPARC Acc L Total | 0.390 | 0.027 |  | Age*SPARC Acc L ML | -0.391 | 0.027 |  | Age*SPARC Acc L ML | -0.418 | 0.017 |
|  | Drugs*SPARC Acc A V | -0.405 | 0.021 |  | ABC*SPARC Acc L V | 0.363 | 0.041 |  | Age*SPARC Acc L Total | -0.368 | 0.038 |  | Age*SPARC Acc L Total | -0.462 | 0.008 |
|  | Drugs*SPARC Acc L AP | -0.366 | 0.039 |  | Age*SPARC Acc A ML | -0.358 | 0.044 |  | Age*SPARC Acc L V | -0.450 | 0.010 |  | Age*SPARC Acc L V | -0.580 | 0.001 |
|  | FES-I*SPARC Acc L ML | -0.382 | 0.031 |  | Age*SPARC Acc A Total | -0.360 | 0.043 |  | LS*SPARC Acc A Total | 0.354 | 0.047 |  | LS*SPARC Acc A AP | 0.388 | 0.028 |
|  | FR*SPARC Acc A AP | 0.422 | 0.016 |  | Age*SPARC Acc A V | -0.397 | 0.025 |  | LS*SPARC Acc L V | 0.378 | 0.033 |  | LS*SPARC Acc A ML | 0.403 | 0.022 |
|  | FR*SPARC Acc A ML | 0.393 | 0.026 |  | Age*SPARC Acc L AP | -0.481 | 0.005 |  |  |  |  |  | LS*SPARC Acc A Total | 0.374 | 0.035 |
|  | FR*SPARC Acc A Total | 0.381 | 0.032 |  | Age*SPARC Acc L ML | -0.392 | 0.026 |  |  |  |  |  | LS*SPARC Acc L AP | 0.396 | 0.025 |
|  | FR*SPARC Acc A V | 0.392 | 0.026 |  | Age*SPARC Acc L Total | -0.372 | 0.036 |  |  |  |  |  | LS*SPARC Acc L ML | 0.383 | 0.031 |
|  | FR*SPARC Acc L AP | 0.361 | 0.043 |  | Age*SPARC Acc L V | -0.422 | 0.016 |  |  |  |  |  | LS*SPARC Acc L Total | 0.371 | 0.036 |
|  | FR*SPARC Acc L ML | 0.403 | 0.022 |  | FR*SPARC Acc A AP | 0.435 | 0.013 |  |  |  |  |  | LS*SPARC Acc L V | 0.474 | 0.006 |
|  | FR*SPARC Acc L Total | 0.404 | 0.022 |  | FR *SPARC Acc A ML | 0.447 | 0.010 |  |  |  |  |  | MMSE*SPARC Acc A AP | 0.487 | 0.005 |
|  | FR*SPARC Acc L V | 0.410 | 0.020 |  | FR *SPARC Acc A Total | 0.449 | 0.010 |  |  |  |  |  | MMSE*SPARC Acc A ML | 0.499 | 0.004 |
|  |  |  |  |  | FR *SPARC Acc A V | 0.452 | 0.009 |  |  |  |  |  | MMSE*SPARC Acc A Total | 0.480 | 0.005 |
|  |  |  |  |  | FR *SPARC Acc L AP | 0.388 | 0.028 |  |  |  |  |  | MMSE*SPARC Acc A V | 0.457 | 0.009 |
|  |  |  |  |  | FR *SPARC Acc L ML | 0.435 | 0.013 |  |  |  |  |  | MMSE*SPARC Acc L AP | 0.434 | 0.013 |
|  |  |  |  |  | FR *SPARC Acc L Total | 0.411 | 0.019 |  |  |  |  |  | MMSE*SPARC Acc L ML | 0.419 | 0.017 |
|  |  |  |  |  | FR *SPARC Acc L V | 0.466 | 0.007 |  |  |  |  |  | MMSE*SPARC Acc L Total | 0.464 | 0.007 |
|  |  |  |  |  | LS *SPARC Acc L V | 0.381 | 0.032 |  |  |  |  |  | MMSE*SPARC Acc L V | 0.388 | 0.028 |
|  |  |  |  |  |  |  |  |  |  |  |  |  |  |  |  |
| Walk 1 | Fallers (single task) |  | |  | Fallers (dual task) |  | |  | Non-fallers (single task) |  | |  | Non-fallers (dual task) |  | |
|  |  | ρ | p |  |  | ρ | p |  |  | ρ | p |  |  | ρ | p |
|  | ABC*SPARC Acc A Total | 0.351 | 0.049 |  | FR *SPARC Acc A Total | 0.454 | 0.009 |  | Age*SPARC Acc A AP | -0.531 | 0.002 |  | Age*SPARC Acc A AP | -0.474 | 0.006 |
|  | Drugs*SPARC Acc A Total | -0.367 | 0.039 |  | FR *SPARC Acc A V | 0.436 | 0.013 |  | Age*SPARC Acc A ML | -0.534 | 0.002 |  | Age*SPARC Acc A ML | -0.472 | 0.006 |
|  | Drugs*SPARC Acc L AP | -0.370 | 0.037 |  | FR *SPARC Acc L AP | 0.411 | 0.019 |  | Age*SPARC Acc A Total | -0.671 | 0.000 |  | Age*SPARC Acc A Total | -0.574 | 0.001 |
|  | FR*SPARC Acc A ML | 0.395 | 0.025 |  | FR *SPARC Acc L ML | 0.389 | 0.028 |  | Age*SPARC Acc A V | -0.600 | 0.000 |  | Age*SPARC Acc A V | -0.455 | 0.009 |
|  | FR*SPARC Acc A Total | 0.438 | 0.012 |  | FR *SPARC Acc L Total | 0.519 | 0.002 |  | Age*SPARC Acc L AP | -0.436 | 0.013 |  | Age*SPARC Acc L AP | -0.584 | 0.000 |
|  | FR*SPARC Acc A V | 0.378 | 0.033 |  | FR *SPARC Acc L V | 0.542 | 0.001 |  | Age*SPARC Acc L ML | -0.460 | 0.008 |  | Age*SPARC Acc L Total | -0.462 | 0.008 |
|  | FR*SPARC Acc L AP | 0.447 | 0.010 |  |  |  |  |  | Age*SPARC Acc L Total | -0.657 | 0.000 |  | Age*SPARC Acc L V | -0.428 | 0.015 |
|  | FR*SPARC Acc L Total | 0.404 | 0.022 |  |  |  |  |  | Age*SPARC Acc L V | -0.486 | 0.005 |  | LS *SPARC Acc A AP | 0.448 | 0.010 |
|  | FR*SPARC Acc L V | 0.418 | 0.017 |  |  |  |  |  | FR*SPARC Acc A Total | 0.459 | 0.008 |  | LS *SPARC Acc A Total | 0.403 | 0.022 |
|  |  |  |  |  |  |  |  |  | FR*SPARC Acc L AP | 0.359 | 0.044 |  | LS *SPARC Acc A V | 0.390 | 0.027 |
|  |  |  |  |  |  |  |  |  | FR*SPARC Acc L Total | 0.373 | 0.035 |  | LS *SPARC Acc L ML | 0.361 | 0.043 |
|  |  |  |  |  |  |  |  |  | FR*SPARC Acc L V | 0.396 | 0.025 |  | LS *SPARC Acc L V | 0.399 | 0.024 |
|  |  |  |  |  |  |  |  |  | LS*SPARC Acc A AP | 0.409 | 0.020 |  | MMSE*SPARC Acc A AP | 0.450 | 0.010 |
|  |  |  |  |  |  |  |  |  | LS*SPARC Acc A ML | 0.387 | 0.029 |  | MMSE*SPARC Acc A ML | 0.385 | 0.030 |
|  |  |  |  |  |  |  |  |  | LS*SPARC Acc A Total | 0.385 | 0.029 |  | MMSE*SPARC Acc A V | 0.453 | 0.009 |
|  |  |  |  |  |  |  |  |  | LS*SPARC Acc A V | 0.378 | 0.033 |  | MMSE *SPARC Acc L AP | 0.351 | 0.049 |
|  |  |  |  |  |  |  |  |  | LS*SPARC Acc L ML | 0.364 | 0.041 |  | MMSE*SPARC Acc L ML | 0.444 | 0.011 |
|  |  |  |  |  |  |  |  |  | LS*SPARC Acc L V | 0.377 | 0.034 |  |  |  |  |
|  |  |  |  |  |  |  |  |  |  |  |  |  |  |  |  |
| Turn | Fallers (single task) |  | |  | Fallers (dual task) |  | |  | Non-fallers (single task) |  | |  | Non-fallers (dual task) |  | |
|  |  | ρ | p |  |  | ρ | p |  |  | ρ | p |  |  | ρ | p |
|  | ABC*SPARC Acc A AP | 0.469 | 0.007 |  | ABC*SPARC Acc A Total | 0.373 | 0.035 |  | ABC*SPARC Acc A AP | 0.364 | 0.041 |  | Age*SPARC Acc A AP | -0.597 | 0.000 |
|  | ABC*SPARC Acc A ML | 0.366 | 0.039 |  | ABC*SPARC Acc A V | 0.376 | 0.034 |  | Age*SPARC Acc A AP | -0.509 | 0.003 |  | Age*SPARC Acc A ML | -0.549 | 0.001 |
|  | ABC*SPARC Acc A Total | 0.468 | 0.007 |  | ABC*SPARC Acc L AP | 0.361 | 0.042 |  | Age*SPARC Acc A ML | -0.456 | 0.009 |  | Age*SPARC Acc A Total | -0.589 | 0.000 |
|  | ABC*SPARC Acc A V | 0.448 | 0.010 |  | FR *SPARC Acc A AP | 0.516 | 0.003 |  | Age*SPARC Acc A Total | -0.610 | 0.000 |  | Age*SPARC Acc A V | -0.602 | 0.000 |
|  | ABC*SPARC Acc L AP | 0.371 | 0.037 |  | FR *SPARC Acc A ML | 0.434 | 0.013 |  | Age*SPARC Acc A V | -0.600 | 0.000 |  | Age*SPARC Acc L AP | -0.551 | 0.001 |
|  | ABC*SPARC Acc L ML | 0.413 | 0.019 |  | FR *SPARC Acc A Total | 0.436 | 0.013 |  | Age*SPARC Acc L AP | -0.477 | 0.006 |  | Age*SPARC Acc L ML | -0.396 | 0.025 |
|  | ABC*SPARC Acc L Total | 0.412 | 0.019 |  | FR *SPARC Acc A V | 0.431 | 0.014 |  | Age*SPARC Acc L Total | -0.428 | 0.015 |  | Age*SPARC Acc L Total | -0.564 | 0.001 |
|  | ABC*SPARC Acc L V | 0.360 | 0.043 |  | FR *SPARC Acc L AP | 0.366 | 0.039 |  | Age*SPARC Acc L V | -0.514 | 0.003 |  | Age*SPARC Acc L V | -0.575 | 0.001 |
|  | Drugs*SPARC Acc L AP | -0.356 | 0.046 |  | FR *SPARC Acc L ML | 0.396 | 0.025 |  | LS*SPARC Acc A V | 0.352 | 0.048 |  | LS *SPARC Acc A AP | 0.373 | 0.036 |
|  | FES-I*SPARC Acc A AP | -0.398 | 0.024 |  | FR *SPARC Acc L Total | 0.413 | 0.019 |  | LS*SPARC Acc L Total | 0.490 | 0.004 |  | LS *SPARC Acc A ML | 0.365 | 0.040 |
|  | FES-I*SPARC Acc L ML | -0.466 | 0.007 |  | FR *SPARC Acc L V | 0.477 | 0.006 |  | LS*SPARC Acc L V | 0.456 | 0.009 |  | LS *SPARC Acc A Total | 0.500 | 0.004 |
|  | FR*SPARC Acc A AP | 0.523 | 0.002 |  |  |  |  |  | MMSE*SPARC Acc L Total | 0.409 | 0.020 |  | LS *SPARC Acc A V | 0.436 | 0.013 |
|  | FR*SPARC Acc A ML | 0.492 | 0.004 |  |  |  |  |  |  |  |  |  | LS *SPARC Acc L AP | 0.426 | 0.015 |
|  | FR*SPARC Acc A Total | 0.556 | 0.001 |  |  |  |  |  |  |  |  |  | LS *SPARC Acc L ML | 0.369 | 0.038 |
|  | FR*SPARC Acc A V | 0.541 | 0.001 |  |  |  |  |  |  |  |  |  | LS *SPARC Acc L Total | 0.440 | 0.012 |
|  | FR*SPARC Acc L AP | 0.523 | 0.002 |  |  |  |  |  |  |  |  |  | LS *SPARC Acc L V | 0.402 | 0.023 |
|  | FR*SPARC Acc L ML | 0.408 | 0.020 |  |  |  |  |  |  |  |  |  | MMSE *SPARC Acc A AP | 0.496 | 0.004 |
|  | FR*SPARC Acc L Total | 0.458 | 0.008 |  |  |  |  |  |  |  |  |  | MMSE*SPARC Acc A ML | 0.472 | 0.006 |
|  | FR*SPARC Acc L V | 0.522 | 0.002 |  |  |  |  |  |  |  |  |  | MMSE*SPARC Acc A Total | 0.523 | 0.002 |
|  |  |  |  |  |  |  |  |  |  |  |  |  | MMSE*SPARC Acc A V | 0.505 | 0.003 |
|  |  |  |  |  |  |  |  |  |  |  |  |  | MMSE*SPARC Acc L AP | 0.531 | 0.002 |
|  |  |  |  |  |  |  |  |  |  |  |  |  | MMSE*SPARC Acc L ML | 0.592 | 0.000 |
|  |  |  |  |  |  |  |  |  |  |  |  |  | MMSE*SPARC Acc L Total | 0.572 | 0.001 |
|  |  |  |  |  |  |  |  |  |  |  |  |  | MMSE*SPARC Acc L V | 0.454 | 0.009 |
|  |  |  |  |  |  |  |  |  |  |  |  |  |  |  |  |
| Walk 2 | Fallers (single task) |  | |  | Fallers (dual task) |  | |  | Non-fallers (single task) |  | |  | Non-fallers (dual task) |  | |
|  |  | ρ | p |  |  | ρ | p |  |  | ρ | p |  |  | ρ | p |
|  | ABC*SPARC Acc A AP | 0.482 | 0.005 |  | ABC*SPARC Acc A AP | 0.392 | 0.026 |  | Age*SPARC Acc A AP | -0.515 | 0.003 |  | Age*SPARC Acc A AP | -0.605 | 0.000 |
|  | ABC*SPARC Acc A ML | 0.392 | 0.026 |  | ABC*SPARC Acc A ML | 0.399 | 0.024 |  | Age*SPARC Acc A ML | -0.579 | 0.001 |  | Age*SPARC Acc A ML | -0.571 | 0.001 |
|  | ABC*SPARC Acc A Total | 0.392 | 0.027 |  | ABC*SPARC Acc A Total | 0.481 | 0.005 |  | Age*SPARC Acc A Total | -0.523 | 0.002 |  | Age*SPARC Acc A Total | -0.571 | 0.001 |
|  | ABC*SPARC Acc A V | 0.465 | 0.007 |  | ABC*SPARC Acc A V | 0.438 | 0.012 |  | Age*SPARC Acc A V | -0.506 | 0.003 |  | Age*SPARC Acc A V | -0.537 | 0.002 |
|  | ABC*SPARC Acc L AP | 0.480 | 0.005 |  | ABC*SPARC Acc L AP | 0.412 | 0.019 |  | Age*SPARC Acc L AP | -0.516 | 0.002 |  | Age*SPARC Acc L AP | -0.449 | 0.010 |
|  | ABC*SPARC Acc L Total | 0.389 | 0.028 |  | Age*SPARC Acc L AP | -0.350 | 0.049 |  | Age*SPARC Acc L Total | -0.467 | 0.007 |  | Age*SPARC Acc L ML | -0.370 | 0.037 |
|  | Drugs*SPARC Acc A Total | -0.395 | 0.025 |  | FR*SPARC Acc A ML | 0.492 | 0.004 |  | Age*SPARC Acc L V | -0.544 | 0.001 |  | Age*SPARC Acc L Total | -0.467 | 0.007 |
|  | Drugs*SPARC Acc A V | -0.357 | 0.045 |  | FR*SPARC Acc A Total | 0.399 | 0.024 |  | FR*SPARC Acc L AP | 0.353 | 0.048 |  | Age*SPARC Acc L V | -0.536 | 0.002 |
|  | Drugs*SPARC Acc L AP | -0.396 | 0.025 |  | FR*SPARC Acc A V | 0.401 | 0.023 |  | LS*SPARC Acc A ML | 0.403 | 0.022 |  | LS*SPARC Acc A AP | 0.416 | 0.018 |
|  | FES-I*SPARC Acc A AP | -0.368 | 0.038 |  | FR*SPARC Acc L AP | 0.377 | 0.033 |  | LS*SPARC Acc A Total | 0.449 | 0.010 |  | LS*SPARC Acc A ML | 0.455 | 0.009 |
|  | FES-I*SPARC Acc L ML | -0.438 | 0.012 |  | FR*SPARC Acc L Total | 0.550 | 0.001 |  | LS*SPARC Acc A V | 0.412 | 0.019 |  | LS*SPARC Acc A Total | 0.511 | 0.003 |
|  | FR*SPARC Acc A ML | 0.479 | 0.006 |  | FR *SPARC Acc L V | 0.413 | 0.019 |  | LS*SPARC Acc L Total | 0.390 | 0.027 |  | LS*SPARC Acc A V | 0.467 | 0.007 |
|  | FR*SPARC Acc A Total | 0.405 | 0.022 |  | LS*SPARC Acc L ML | 0.351 | 0.049 |  | MMSE*SPARC Acc A AP | 0.357 | 0.045 |  | LS*SPARC Acc L AP | 0.355 | 0.046 |
|  | FR *SPARC Acc A V | 0.368 | 0.038 |  | LS *SPARC Acc L V | 0.356 | 0.046 |  | MMSE*SPARC Acc A Total | 0.377 | 0.033 |  | LS*SPARC Acc L ML | 0.405 | 0.022 |
|  | FR*SPARC Acc L Total | 0.561 | 0.001 |  |  |  |  |  | MMSE*SPARC Acc A V | 0.364 | 0.041 |  | LS*SPARC Acc L V | 0.376 | 0.034 |
|  |  |  |  |  |  |  |  |  |  |  |  |  | MMSE*SPARC Acc A AP | 0.524 | 0.002 |
|  |  |  |  |  |  |  |  |  |  |  |  |  | MMSE*SPARC Acc A ML | 0.471 | 0.007 |
|  |  |  |  |  |  |  |  |  |  |  |  |  | MMSE*SPARC Acc A Total | 0.493 | 0.004 |
|  |  |  |  |  |  |  |  |  |  |  |  |  | MMSE*SPARC Acc A V | 0.570 | 0.001 |
|  |  |  |  |  |  |  |  |  |  |  |  |  | MMSE*SPARC Acc L AP | 0.570 | 0.001 |
|  |  |  |  |  |  |  |  |  |  |  |  |  | MMSE*SPARC Acc L ML | 0.486 | 0.005 |
|  |  |  |  |  |  |  |  |  |  |  |  |  | MMSE*SPARC Acc L Total | 0.435 | 0.013 |
|  |  |  |  |  |  |  |  |  |  |  |  |  |  |  |  |
| Turn to sit | Fallers (single task) |  | |  | Fallers (dual task) |  | |  | Non-fallers (single task) |  | |  | Non-fallers (dual task) |  | |
|  |  | ρ | p |  |  | ρ | p |  |  | ρ | p |  |  | ρ | p |
|  | ABC*SPARC Acc A AP | 0.363 | 0.041 |  | ABC*SPARC Acc A AP | 0.355 | 0.046 |  | LS*SPARC Acc A ML | 0.582 | 0.000 |  | SBP*SPARC Acc A V | -0.403 | 0.022 |
|  | ABC*SPARC Acc A ML | 0.436 | 0.013 |  | ABC*SPARC Acc A V | 0.459 | 0.008 |  | LS*SPARC Acc A Total | 0.448 | 0.010 |  | FES-I*SPARC Acc A AP | 0.354 | 0.047 |
|  | ABC*SPARC Acc A V | 0.381 | 0.031 |  | Age*SPARC Acc A AP | -0.397 | 0.024 |  | LS*SPARC Acc L AP | 0.390 | 0.027 |  | FES-I*SPARC Acc A ML | 0.385 | 0.030 |
|  | ABC*SPARC Acc L AP | 0.354 | 0.047 |  | Age*SPARC Acc A Total | -0.368 | 0.038 |  | LS*SPARC Acc L Total | 0.501 | 0.003 |  | LS*SPARC Acc A ML | 0.399 | 0.024 |
|  | ABC*SPARC Acc L ML | 0.394 | 0.026 |  | Age*SPARC Acc A V | -0.360 | 0.043 |  | LS*SPARC Acc L V | 0.453 | 0.009 |  | LS*SPARC Acc A V | 0.367 | 0.039 |
|  | Drugs*SPARC Acc A Total | -0.352 | 0.048 |  | Age*SPARC Acc L AP | -0.482 | 0.005 |  | MMSE*SPARC Acc A AP | 0.404 | 0.022 |  | MAP*SPARC Acc A V | -0.408 | 0.020 |
|  | Drugs*SPARC Acc A V | -0.424 | 0.016 |  | Drugs*SPARC Acc A V | -0.401 | 0.023 |  | MMSE*SPARC Acc A ML | 0.519 | 0.002 |  |  |  |  |
|  | Drugs*SPARC Acc L AP | -0.370 | 0.037 |  | Drugs*SPARC Acc L V | -0.351 | 0.049 |  | MMSE*SPARC Acc A Total | 0.411 | 0.020 |  |  |  |  |
|  | FR *SPARC Acc A V | 0.427 | 0.015 |  | FR *SPARC Acc A V | 0.401 | 0.023 |  | MMSE*SPARC Acc L AP | 0.355 | 0.046 |  |  |  |  |
|  |  |  |  |  |  |  |  |  | MMSE*SPARC Acc L Total | 0.386 | 0.029 |  |  |  |  |
|  |  |  |  |  |  |  |  |  | MMSE*SPARC Acc L V | 0.355 | 0.046 |  |  |  |  |
|  |  |  |  |  |  |  |  |  |  |  |  |  |  |  |  |
| Full TUG | Fallers (single task) |  | |  | Fallers (dual task) |  | |  | Non-fallers (single task) |  | |  | Non-fallers (dual task) |  | |
|  |  | ρ | p |  |  | ρ | p |  |  | ρ | p |  |  | ρ | p |
|  | ABC*SPARC Acc A AP | 0.397 | 0.024 |  | ABC*SPARC Acc A AP | 0.398 | 0.024 |  | Age*SPARC Acc A AP | -0.456 | 0.009 |  | Age*SPARC Acc A ML | -0.430 | 0.014 |
|  | ABC*SPARC Acc A ML | 0.429 | 0.014 |  | ABC*SPARC Acc A ML | 0.474 | 0.006 |  | Age*SPARC Acc A ML | -0.468 | 0.007 |  | Age*SPARC Acc A Total | -0.475 | 0.006 |
|  | ABC*SPARC Acc A Total | 0.362 | 0.042 |  | ABC*SPARC Acc A Total | 0.412 | 0.019 |  | Age*SPARC Acc A Total | -0.464 | 0.008 |  | Age*SPARC Acc A V | -0.400 | 0.023 |
|  | ABC*SPARC Acc L AP | 0.460 | 0.008 |  | ABC*SPARC Acc L AP | 0.429 | 0.014 |  | Age*SPARC Acc A V | -0.459 | 0.008 |  | Age*SPARC Acc L AP | -0.399 | 0.024 |
|  | ABC*SPARC Acc L V | 0.424 | 0.016 |  | ABC*SPARC Acc L Total | 0.424 | 0.016 |  | Age*SPARC Acc L AP | -0.456 | 0.009 |  | Age*SPARC Acc L ML | -0.454 | 0.009 |
|  | FR*SPARC Acc A AP | 0.370 | 0.037 |  | ABC*SPARC Acc L V | 0.453 | 0.009 |  | Age*SPARC Acc L ML | -0.409 | 0.020 |  | Age*SPARC Acc L Total | -0.417 | 0.018 |
|  | FR*SPARC Acc A ML | 0.483 | 0.005 |  | FR*SPARC Acc A V | 0.359 | 0.044 |  | Age*SPARC Acc L V | -0.571 | 0.001 |  | Age*SPARC Acc L V | -0.475 | 0.006 |
|  | FR*SPARC Acc A Total | 0.491 | 0.004 |  |  |  |  |  | LS*SPARC Acc L V | 0.415 | 0.018 |  | FR*SPARC Acc A ML | 0.356 | 0.046 |
|  | FR*SPARC Acc A V | 0.422 | 0.016 |  |  |  |  |  |  |  |  |  | FR*SPARC Acc A Total | 0.425 | 0.015 |
|  | FR*SPARC Acc L AP | 0.409 | 0.020 |  |  |  |  |  |  |  |  |  | MMSE*SPARC Acc L V | 0.351 | 0.049 |
|  | FR*SPARC Acc L Total | 0.490 | 0.004 |  |  |  |  |  |  |  |  |  |  |  |  |
|  | FR*SPARC Acc L V | 0.494 | 0.004 |  |  |  |  |  |  |  |  |  |  |  |  |
| Note: only significant correlations are shown (Spearman correlation, p < 0.05). FES-I: The Falls Efficacy Scale International; ABC: Activities-Specific Balance Confidence Scale; FR: Functional Reach test; MMSE: Mini-mental mini mental state examination; LS: Level of schooling; SBP: systolic blood pressure; MAP: mean arterial pressure. | | | | | | | | | | | | | | | |
